# The Murine Gammaherpesvirus Immediate-Early Rta Synergizes with IRF4, Targeting Expression of the Viral M1 Superantigen to Plasma Cells

**DOI:** 10.1371/journal.ppat.1004302

**Published:** 2014-08-07

**Authors:** Brigid M. O'Flaherty, Tanushree Soni, Brian S. Wakeman, Samuel H. Speck

**Affiliations:** 1 Department of Microbiology and Immunology, Emory University School of Medicine, Atlanta, Georgia, United States of America; 2 Emory Vaccine Center, Emory University School of Medicine, Atlanta, Georgia, United States of America; University of North Carolina at Chapel Hill, United States of America

## Abstract

MHV68 is a murine gammaherpesvirus that infects laboratory mice and thus provides a tractable small animal model for characterizing critical aspects of gammaherpesvirus pathogenesis. Having evolved with their natural host, herpesviruses encode numerous gene products that are involved in modulating host immune responses to facilitate the establishment and maintenance of lifelong chronic infection. One such protein, MHV68 M1, is a secreted protein that has no known homologs, but has been shown to play a critical role in controlling virus reactivation from latently infected macrophages. We have previous demonstrated that M1 drives the activation and expansion of Vβ4^+^ CD8^+^ T cells, which are thought to be involved in controlling MHV68 reactivation through the secretion of interferon gamma. The mechanism of action and regulation of M1 expression are poorly understood. To gain insights into the function of M1, we set out to evaluate the site of expression and transcriptional regulation of the M1 gene. Here, using a recombinant virus expressing a fluorescent protein driven by the M1 gene promoter, we identify plasma cells as the major cell type expressing M1 at the peak of infection in the spleen. In addition, we show that M1 gene transcription is regulated by both the essential viral immediate-early transcriptional activator Rta and cellular interferon regulatory factor 4 (IRF4), which together potently synergize to drive M1 gene expression. Finally, we show that IRF4, a cellular transcription factor essential for plasma cell differentiation, can directly interact with Rta. The latter observation raises the possibility that the interaction of Rta and IRF4 may be involved in regulating a number of viral and cellular genes during MHV68 reactivation linked to plasma cell differentiation.

## Author Summary

Through coevolution with their hosts, gammaherpesviruses have acquired unique genes that aid in infection of a particular host. Here we study the regulation of the MHV68 M1 gene, which encodes a protein that modulates the host immune response. Using a strategy that allowed us to identify MHV68 infected cells in mice, we have determined that M1 expression is largely limited to the antibody producing plasma cells. In addition, we show that M1 gene expression is regulated by both cellular and viral factors, which allow the virus to fine-tune gene expression in response to environmental signals. These findings provide insights into M1 function through a better understanding of how M1 expression is regulated.

## Introduction

MHV68 is a naturally occurring murid gammaherpesvirus that has significant genetic and functional homology to the human gammaherpesviruses Epstein-Barr virus (EBV) and Kaposi's sarcoma-associated herpesvirus (KSHV). Among herpesviruses, there are a large number of genes involved in virus replication that are conserved – both in sequence and spatial arrangement in the viral genome. However, every herpesvirus, having co-evolved with its host during speciation, has acquired unique genes - many of which function to modulate and/or evade the host immune response. Coevolution of with their hosts has led to some divergence of host-pathogen interactions; however, unique genes may reveal homologous functions required for chronic infection of the host. One such gene is the MHV68 M1, which is found in a cluster of unique genes at the left end of the MHV68 genome.

Initial functional studies of M1, utilizing an M1-null virus revealed a hyper-reactivation phenotype from latently infected peritoneal exudate cells (PEC) [1]. Subsequent studies found that this hyper-reactivation phenotype was strain specific – occurring in C57Bl/6 mice, but not Balb/c mice [2]. In addition to the strain specific reactivation phenotype, a strain specific expansion of Vβ4^+^CD8^+^ T cells had previously been observed in response to MHV68 infection [3]. This pronounced T cell expansion and activation is a hallmark of MHV68 infection in many inbred mouse strains and is observed in peripheral lymphoid organs, as well as the blood, reaching peak levels after the virus has established latency [3], [4]. Notably, the Vβ4^+^CD8^+^ T cells remain elevated during the course of chronic MHV68 infection, and do not adopt an exhausted phenotype [3]. Analysis of M1-null mutants revealed that a functional M1 gene is required for the Vβ4^+^CD8^+^ T cell expansion [2]. Furthermore, M1 was shown to be a secreted protein capable of stimulating Vβ4^+^CD8^+^ T cells to produce IFNγ and TNFα [2]. These analyses suggested that M1 may exert control over MHV68 reactivation from peritoneal macrophages through the induction of IFNγ from Vβ4^+^CD8^+^ T cells [2], this is supported by the observations that: (i) IFNγ−/− mice exhibit hyper-reactivation from PECS [5]; and (ii) the demonstration that IFNγ can suppress MHV68 replication in macrophages [2], [6], [7].

Early experiments to evaluate the expansion in thymectomized mice suggested that Vβ4^+^CD8^+^ T cells are maintained through continued stimulation by a stimulatory ligand, which is now known to be M1 [8]. Interestingly, B cells appear to play a critical role in the expansion of Vβ4^+^CD8^+^ T cells, as no expansion is observed upon MHV68 infection of mice lacking B cells [9], [10]. Other studies provide some clues to the timing and site of M1 expression during MHV68 infection, where B220^+^ splenocytes at 14 days post-infection were found to be capable of stimulating Vβ4^+^CD8^+^ T cell hybridomas [11].

Though no homolog to M1 has been found in other gammaherpseviruses, HVS has been shown to encode a viral superantigen, immediate early gene ie14/vsag [12]. Like M1, ie14/vsag, is not essential for viral replication; and interestingly, ie14/vsag expression is elevated in phorbol ester treated cells, indicating a link with viral reactivation. In EBV, structural protein gp350, as well as latent membrane proteins LMP-1 and LMP-2A have been shown to activate expression of an endogenous human retroviral superantigen, HERV-K18, which results in a Vβ13^+^ T cell expansion [13]–[15]. Due to limitations in study of non-human primate and human patients it has been difficult to assess the role of these superantigens and the consequence of their resulting T cell expansion. We are therefore left to speculate what benefit they provide to their host. Do they aid in infection or the establishment of latency? Do they divert the immune response? Are they involved in control of infection? We hope that a better understanding of the expression and role of M1 in MHV68 infection may shed light into the conserved use of viral superantigens by gammaherpseviruses.

Though numerous studies to define the transcriptional program of MHV68 *in vitro* have identified M1 as an early through late gene [16]–[18] relatively little is known about when and where M1 is expressed during infection. Furthermore, while a number of transcriptome based analyses have detected transcripts extending through the M1 locus during *in vivo* infection, much of this data relies on methods that are not strand specific and therefore not definitive [19]–[22]. Due to the dearth of information about M1expression *in vivo*, we set out to characterize M1 expression using a novel approach wherein a fluorescent reporter virus would allow detection of M1 promoter activity during infection. This approach led to the identification of splenic plasma cells as the primary cell type expressing M1 *in vivo*. Furthermore, factors regulating M1 transcription were previously uncharacterized. The current studies have elucidated key cis-elements and transcription factors controlling the expression of M1 in plasma cells. Overall, these findings provide insights into the role of M1-mediated regulation of MHV68 pathogenesis. Moreover, we reveal a novel and potentially conserved mechanism which controls the timing and site of viral gene expression in response to reactivation in the B cell.

## Results

### M1 is expressed in a subset of MHV68 infected splenocytes

**Figure 1:**
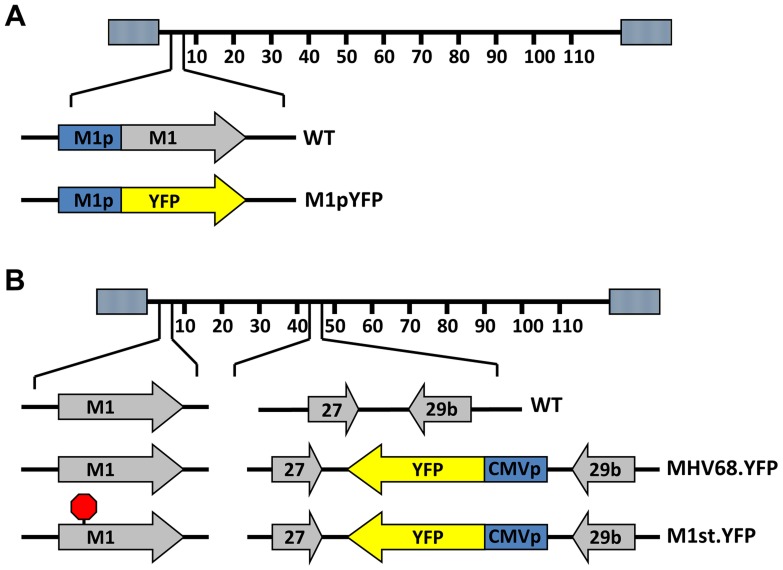
Generation of YFP reporter viruses. C57Bl/6 mice were intranasally infected with 5×10^5^ pfu of the indicated virus and spleens were harvested at 14 days post infection. (A) To assess M1 promoter activity, a YFP cassette was cloned in place of the M1 open reading frame, allowing marking of infected cells where M1 promoter was active. (B) To determine MHV68 infection in the absence of M1 expression, a translational stop codon was introduced into the M1 open reading frame (ORF) of the MHV68-YFP BAC [23] by allelic exchange as previously described [2].

To identify cellular reservoirs in which the M1 gene is expressed *in vivo*, we generated a series of recombinant viruses that express yellow fluorescent protein (YFP) to mark infected cells. For detection of M1 promoter activity, the M1 coding sequence was replaced with that encoding YFP, creating a M1 promoter-driven YFP mutant (Figure 1A). This strategy allows detection of the cellular reservoirs in which M1 is expressed during infection. Additionally, two important controls were used: MHV68-YFP, in which the YFP transgene under the control of the HCMV IE promoter was cloned into a neutral locus in the viral genome (efficiently marking MHV68 infected B cells and plasma cells) [23]; and (ii) MHV68-M1st.YFP, which contains the M1 translational stop mutation (M1-null virus) in the context of the YFP transgene cloned into the neutral locus (Figure 1B). As M1 has previously been identified as a non-essential for both virus replication and for the establishment of latency *in vivo* [24], we did not anticipate that the M1pYFP recombinant would change the cellular reservoirs infected by MHV68. However, to formally address this issue, we have included analyses of the MHV68-M1st.YFP virus – which like the M1pYFP lacks a functional M1 gene.

**Figure 2:**
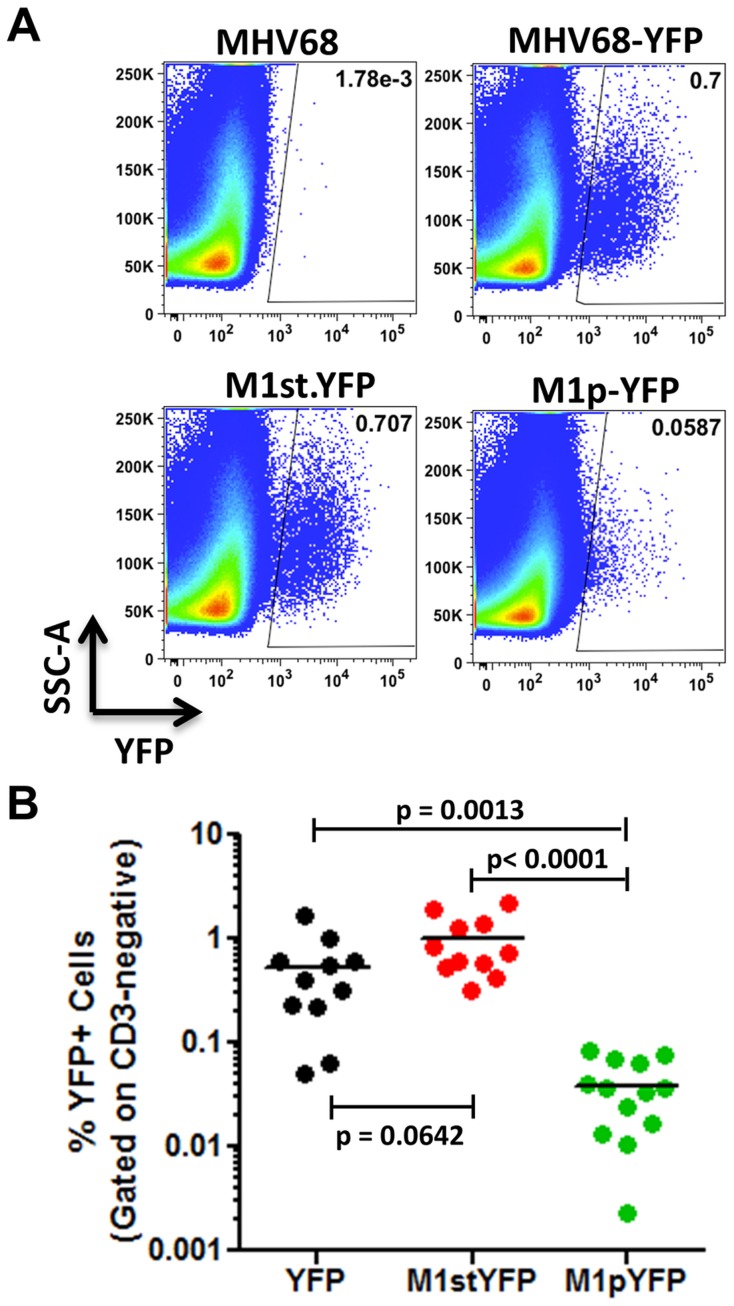
M1 promoter activity is detected in a subset of MHV68 infected splenocytes. C57Bl/6 mice were intranasally infected with 5×10^5^ pfu of the indicated virus and spleens were harvested at 14 days post infection. Splenocytes were gated on the CD3-negative fraction to eliminate auto-fluorescing cells, as previously described [23]. (A) Representative plots show YFP marking of infected splenocytes. YFP-positive gates were based on mice infected with wild type MHV68 lacking a YFP expression cassette. (B) Complied results from 3 experiments, with 3–5 mice per group, show the frequency of YFP^+^ cells in spleens of infected mice.

Analysis of MHV68 infection of splenocytes at day 14 post-infection revealed robust marking of splenocytes by both the MHV68-YFP and MHV68-M1stYFP viruses (Figure 2). We have previously noted that there is significant mouse to mouse variation in the frequency of infected splenocytes for a given virus [25], and have recently determined that this directly correlates with the frequency of the CD4^+^ T follicular helper (T_FH_) response [26]. For these analyses we observed on average ca. 0.5% and 1.0% of splenocytes were YFP^+^ for the MHV68-YFP and MHV68-M1stYFP viruses, respectively (Figure 2). The latter result confirms that M1 function is dispensable for the establishment of latency in splenocytes. In contrast, only ca. 0.04% of splenocytes were YFP^+^ with the M1pYFP virus, indicating that the M1 promoter is active in only ca. 5–10% of infected splenocytes.

### The majority of M1 expression is detected in MHV68 infected plasma cells

**Figure 3:**
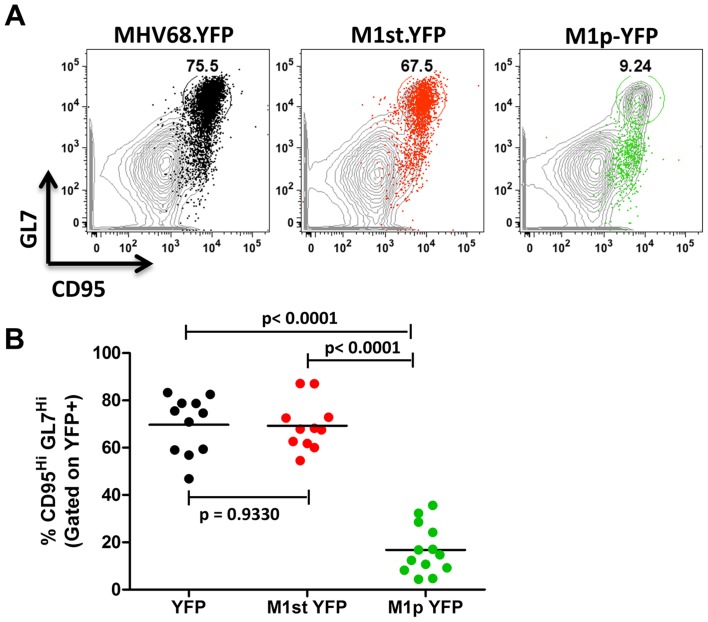
Low levels of M1 promoter activity are detected in germinal center B cells. C57Bl/6 mice were intranasally infected with 5×10^5^ pfu of the indicated virus and spleens were harvested at 14 days post infection. B cells were defined by CD19^+^CD3^−^ population. (A) Representative plots show YFP marking (colored) overlayed on total B cell population (gray) and are gated for germinal center B cells defined by GL7^Hi^CD95^Hi^. (B) Complied results from 3 experiments, with 3–5 mice per group, show the frequency of YFP^+^ cells with a germinal center B cell phenotype.

We have previously shown that the majority (ca. 70–90%) of virally infected B cells, as indicated by YFP expression, exhibit a germinal center phenotype [23], [27]. Individual mice were assessed for YFP marking and, consistent with previous observations, we found a similar frequency of virus infected (YFP^+^) B cells with a germinal center phenotype for mice infected with either the MHV68.YFP or MHV68-M1st.YFP viruses, both showing an average of ca. 70% (Figure 3). These results further substantiate that a functional M1 gene is dispensable for establishment of MHV68 latency in B cells. In contrast, few infected germinal center B cells were marked by the M1pYFP virus (an average of ca. 20% of YFP^+^ cells) – indicating that the majority of M1 expressing cells do not have a germinal center B cell phenotype. Based on the ca. 10-fold lower frequency of splenocytes marked by the M1pYFP virus (see Figure 2), we estimate that M1 promoter activity is only detectable in ca. 5% of infected germinal center B cells. Based on these results it is clear that M1 is predominantly expressed in some other MHV68 infected cellular reservoir.

**Figure 4:**
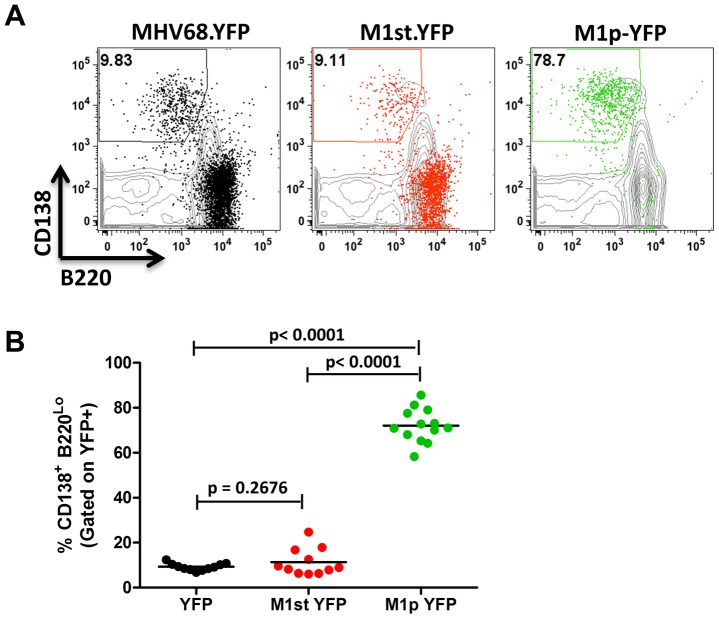
The majority of M1 promoter activity is detected in splenic plasma cells. C57Bl/6 mice were infected with 5×10^5^ pfu/IN of the indicated virus and spleens were harvested at 14 days post infection. Cells were gated on CD3^−^ population for analysis. (A) Representative plots show YFP marking (colored) overlayed on total CD3^−^ population (gray) and are gated for plasma cells defined by CD138^+^B220^Lo^. (B). Compiled results from 3 experiments, with 3–5 mice per group, show the frequency of YFP^+^ cells with a plasma cell phenotype.

The other major cell population in the spleen that is infected by MHV68 are plasma cells (CD138^hi^, B220^low^) [23], [27]. During infection, virus infection (YFP marking) of splenic plasma cells reaches peak levels at day 14 post-infection (ca. 10–20% of virus infected splenocytes) and begins to wane by day 18 post-infection (ca. 5–10% of virus infected splenocytes) [23]. We observed marking of splenic plasma cells for both MHV68-YFP and MHV68-M1st.YFP infected mice at day 14 post-infection consistent with previous observations, with ca. 10% YFP^+^ cells exhibiting a plasma cell phenotype (no significant difference between these 2 groups) (Figure 4). Strikingly, when assessing YFP marking of the splenic plasma cell population by the M1pYFP virus, the vast majority of YFP+ cells exhibited a plasma cell phenotype (on average >75% of YFP+ cells) (Figure 4). Thus, this strongly argues that M1 gene expression is largely limited to the infected plasma cell population. Notably, MHV68 reactivation from latently infected splenocytes is tightly linked to plasma cell differentiation [27], which suggests that M1 expression is coupled to virus reactivation from B cells. Finally, when considering the frequency of M1pYFP marked cells with the frequency of MHV68-YFP and MHV68-M1st.YFP marked splenic plasma cells, it appears that the majority of virus infected plasma cells express M1.

### Identification and initial characterization of the M1 gene promoter

**Figure 5:**
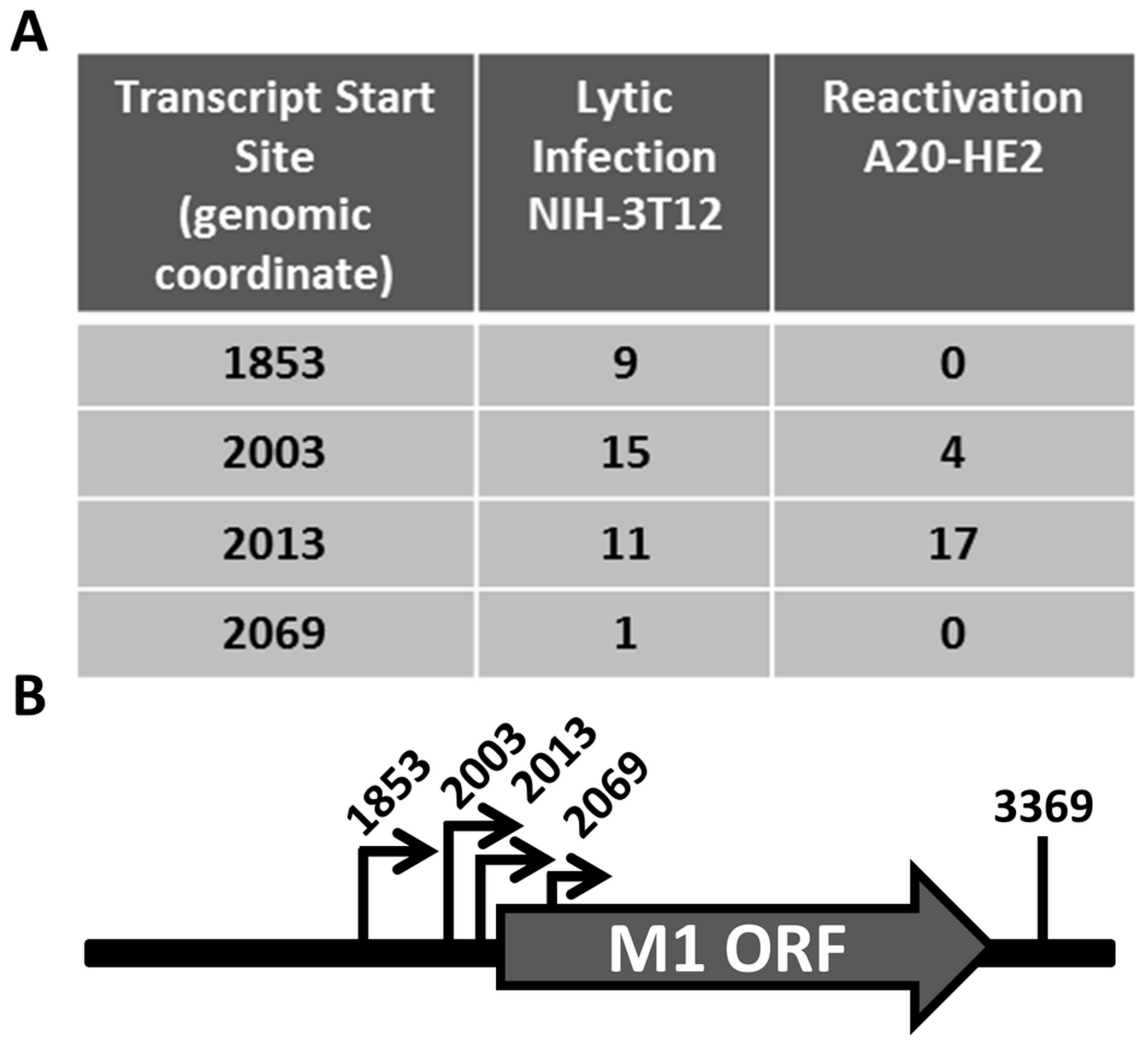
M1 transcript mapping identifies initiation and termination sites which result in a 1.3 and 1.5 (A) 5′ and 3′ rapid amplification of cDNA ends (RACE) was performed using RNA isolated from infected NIH3T12 fibroblasts and stimulated MHV68 infected A20-HE2 cells. The number of RACE clones that were identified for transcript initiation and termination sites are summarized. (B) A diagram of the transcript initiation and termination sites is shown with the genomic coordinates.

Having identified the reservoir where M1 is expressed *in vivo*, we sought to characterize the structure of the M1 transcript and to identify the M1 promoter. Rapid amplification of cDNA ends (RACE) was done to identify the transcript initiation and termination sites in two cell lines: (i) infected NIH3T12 fibroblasts; and (ii) reactivated A20-HE2 cells. A20-HE2 cells are a stable lymphoblast B cell line which carry the MHV68 genome where viral reactivation can be induced by tetradecanoylphorbol acetate (TPA) [28]. RNA and protein were collected from both cell lines and lytic gene expression was confirmed prior to analysis (data not shown). Transcript analysis revealed four initiation sites and a single termination site from an unspliced transcript (Figure 5A, 5B). Though all transcript initiation sites were found in infected 3T12 cells, only transcripts starting at bp 2003 and bp 2013 were detected from reactivated A20-HE2 cells. The sizes of the predicted unspliced M1 transcripts were confirmed by northern analyses of RNA prepared from: (i) TPA stimulated A20-HE2 cells (a MHV68 latently infected B cells); and (ii) MHV68 infected NIH 3T12 fibroblasts (data not shown).

**Figure 6:**
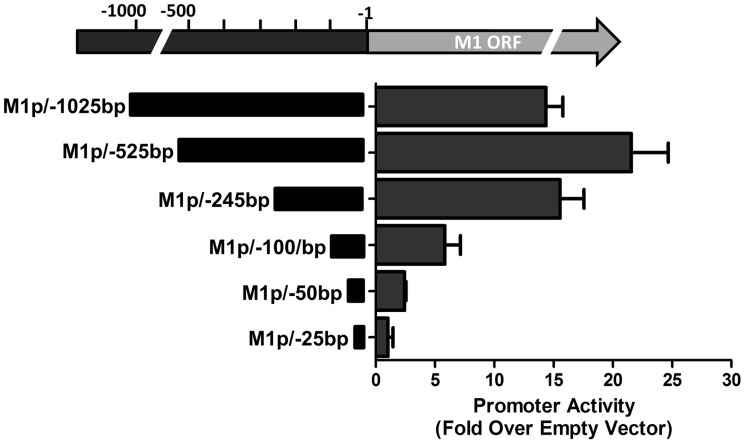
M1 promoter exhibits basal activity in a plasmablast cell line. Serial truncations of the M1 promoter region were cloned into a luciferase reporter vector and tested for luciferase activity in the P3X63Ag8 cell line from ATCC. P3X63Ag8 cells were nucleofected and 48(pGL4.10). Experiments were done with triplicate samples and repeated in two independent experiments.

To identify the regulatory elements controlling M1 gene expression we next set out to characterize the M1 promoter. Serial truncations of the putative M1 promoter region were cloned into a luciferase reporter vector and tested for promoter activity in a variety of cell lines. Notably, minimal activity was detected in the murine B cell lines A20, WEHI, NSO, and BCL1-3B3 (data not shown) – perhaps consistent with the failure to observe significant M1 promoter-driven YFP activity in most splenic B cell populations with the MHV68-M1pYFP virus in mice. In addition, we failed to detect significant activity from these reporter constructs in the murine macrophage cell line RAW264.7 (data not shown). However, when these reporter constructs were transfected into the P3X68Ag8 murine plasmacytoma cell line significant basal promoter activity was observed (Figure 6). Similar levels of M1 promoter-driven luciferase activity were observed for the longer M1 promoter constructs (M1p/−1025 bp, M1p/−525 bp, and M1p/−245 bp), while truncation of sequences upstream of −100 bp significantly decreased activity (Figure 6). Activity was further decreased to near background levels when sequences upstream of −50 bp were deleted (Figure 6).

The region upstream of the M1 transcription initiation sites was screened for the presence of candidate transcription factor binding sites [University of Pennsylvania Transcription Element Search System (TESS)]. TESS and manual sequence analyses identified a number of candidate transcription factor binding sites for NFκB, GATA3, IRF8/IRF4, and RBPJκ. Because M1 promoter activity was detected in plasma cells *in vivo*, interferon regulatory factor 4 (IRF4), a transcription factor upregulated in plasma cells which plays a critical role in plasma cell differentiation as well as immunoglobulin class switch recombination ([29]–[31] and reviewed in [32]), was of particular interest.

**Figure 7:**
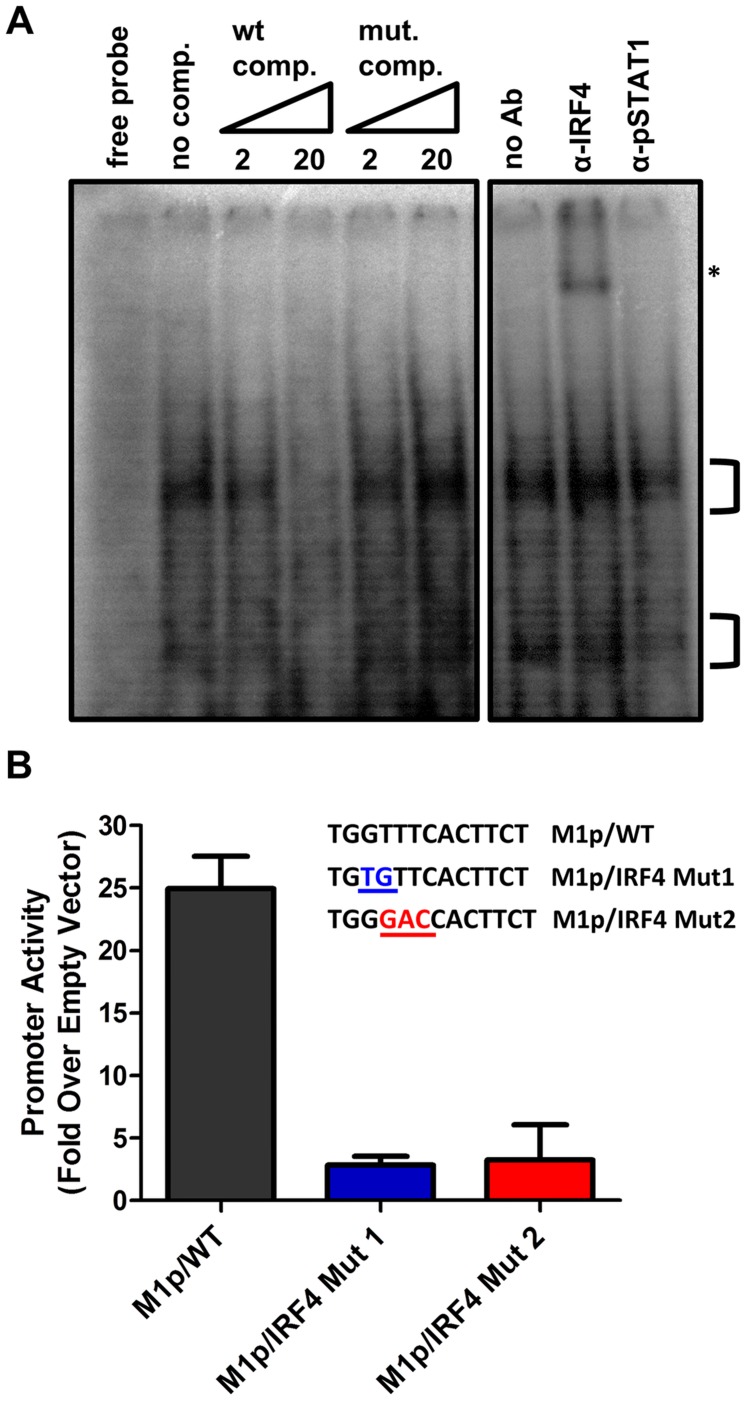
Basal activity of the M1 promoter is dependent on IRF4 binding. (A) To identify DNA-protein interaction an electrophoretic mobility shift assay was performed using radiolabeled probe with P3X nuclear extracts. A competition assay to determine the specificity of DNA-protein complexes used 2 and 20 fold molar excess of unlabeled competitor DNA containing either the WT or mutant IRF4 binding sequence. An antibody supershift assay was performed by preincubating samples with anti-IRF4, or an isotype control antibody (anti-pSTAT1) prior to electrophoresis. The asterisk denotes the anti-IRF4 supershifted complex. (B) Point mutations in the M1 promoter were made in the IRF4 binding site and assessed in the P3X63Ag8 plasmacytoma cell line.

To characterize IRF4 binding to the candidate IRF site in the M1 promoter, an electrophoretic mobility shift assay (EMSA) was carried out (Figure 7A). EMSA was performed using nuclear extracts from P3X63Ag8 cells grown under normal conditions, along with a [^32^P]-labeled oligonucleotide probe containing the candidate M1 promoter IRF4 binding site. As expected we observed shifted complexes, which could be competed away using unlabeled double stranded DNA probes containing the M1p IRF4 binding site, but not with a competitor containing an IRF binding site mutation which has previously been shown to disrupt IRF8 binding with DNA [33] (Figure 7A). Furthermore, binding of IRF4 was confirmed by supershift analysis using an antibody against IRF4 (Figure 7A). This analysis was extended by generating M1 promoter-driven luciferase reporter constructs in which mutations were introduced into the IRF binding site. Two mutations in the core interferon response sequence, which have previously been shown to ablate IRF8 DNA:protein interaction [33], were introduced into the M1 promoter. Notably, either mutation led to a significant loss in basal M1 promoter activity (ca. 8-fold decrease in promoter activity) (Figure 7B).

### Viral Rta and cellular IRF4 synergize to activate transcription from the M1 promoter

**Figure 8:**
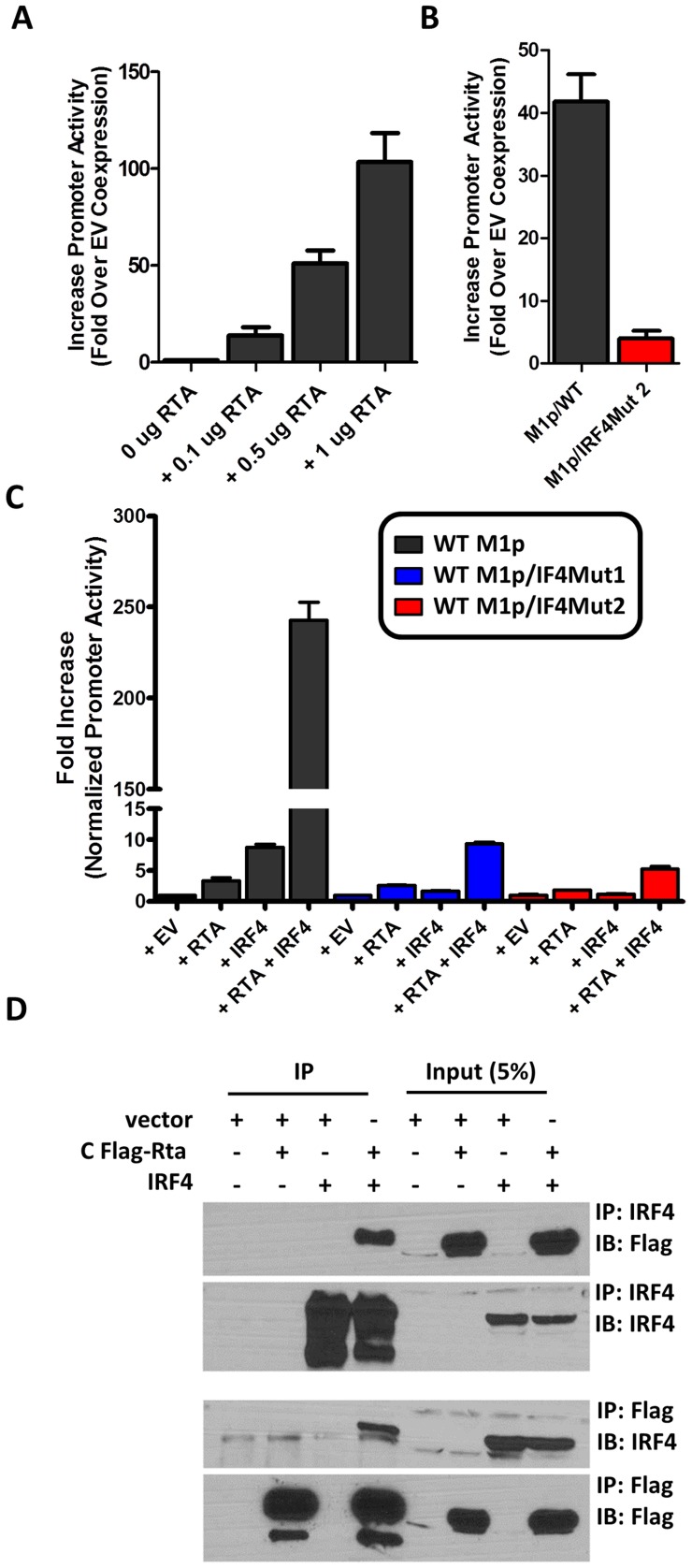
Efficient RTA transactivation of M1 promoter is dependent on a functional IRF4 binding site. (A) To evaluate the ability of Rta to transactivate the M1 promoter, a dosage response using increasing amounts of Rta expression vector war performed in P3X63Ag8 cells. (B) Mutations to the IRF4 binding motif were then generated and tested for the ability to respond to Rta in P3X63Ag8 cells. (C) Synergistic effects of RTA and IRF4 on WT and mutated M1 promoter were evaluated by expressing Rta or IRF4 alone or in combination (normalized with pCDNA empty vector) in 293T cells. (D) Lysates from 293T cells transfected with Rta or IRF4 alone or in combination were used for co-immunoprecipitation with IRF4 or Flag antibody. Membranes were probed with the anti-Flag or anti-IRF4 to for detection. The data shown for the reporter gene assays (panels A–C) were done with triplicate samples, and were repeated in 2–3 independent experiments. Standard error of the mean is shown.

Several studies have established a link between gammaherpesvirus reactivation from latency and plasma cell differentiation [27], [34]–[39]. Given that our data shows: (i) M1 promoter expression is detected from plasma cells during *in vivo* infection; (ii) basal M1 promoter activity requires a functional IRF4 site; and (iii) viral reactivation is linked with plasma cell differentiation, we set out to evaluate whether the M1 promoter is responsive to the MHV68 viral lytic transactivator Rta. Expressing increasing amounts of Rta with an M1 promoter-driven reporter construct in the P3X63Ag8 plasmacytoma cell line resulted in a dosage dependent increase in M1 promoter activity (Figure 8A). Moreover, the ability of Rta to efficiently transactivate the M1 promoter in the P3X63Ag8 cell line was dependent on the presence of an intact IRF4 binding site (Figure 8B). To further assess whether Rta functionally synergizes with IRF4 to activate the M1 promoter, we chose a cell line (293T cells) which lacks expression of Rta and IRF4. In 293T cells we observed that either factor alone led to very modest increase in M1 promoter activity (ca. 5–10 fold) (Figure 8C). However, when the two factors were co-expressed there was a significant increase in promoter activity (ca. 250-fold) (Figure 8C). Importantly, disruption of the IRF4 binding site dramatically impaired the ability of IRF4 and Rta to synergistically activate the M1 promoter (Figure 8C).

Based on the synergy between Rta and IRF4 in activating the M1 promoter, we assessed whether these factors can physically interact with each other. A co-immunoprecipitation was performed with cell lysates from transfected 293T cells. Immunoprecipitation with anti-IRF4 antibody, followed by anti-Flag detection of Rta, resulted in detection of a 90 kD band corresponding to Rta that was present only when Rta and IRF4 were co-expressed in 293T cells (Figure 8D). Following detection of Rta the blot was stripped and probed for IRF4 to confirm expression of the 52 kD band corresponding to IRF4. IRF4 was detected in whole cell lysates and immunoprecipitated samples containing IRF4. The reciprocal blot using anti-flag for immunoprecipitation and anti-IRF4 for detection showed a 52 kD band corresponding to IRF4. Additionally, Rta was detected from whole cell lysates and immunoprecipitated samples containing Rta. These results are consistent with a physical interaction between Rta and IRF4 that likely facilitates that observed synergy of these factors in activating M1 gene expression.

**Figure 9:**
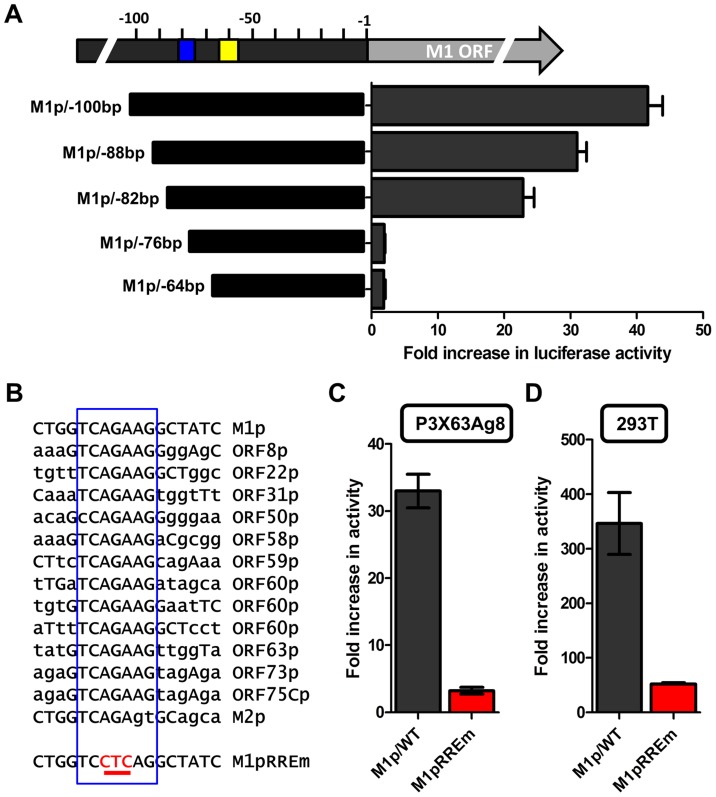
Identification of a novel RTA response element in the M1 promoter. (A) To identify the Rta response element serial truncations of the M1 promoter were tested for RTA transactivation in the P3X63Ag8 plasmacytoma line which constitutively expresses IRF4. The blue box indicates the putative RTA response element; the yellow box denotes the location of the IRF4 binding site. (B) The nucleotide sequence of the putative Rta response element found in the M1 promoter is shown, along with nucleotide sequences sharing the core sequence with the M1 Rta response element (RRE) (denoted by the open blue rectangle) that are located upstream of a number of other MHV68 genes. In addition, the mutation (M1pRREm) introduced into the M1 Rta response element is shown. (C and D) Activity of the M1 promoter-driven reporter construct containing a mutation (indicated in red) in the putative Rta response element (RREm) was assessed in the P3X63Ag8 plasmacytoma cell line for Rta transactivation (panel C) and in 293T cells for IRF4+Rta synergy (panel D). Transient transfection reporter gene assays were conducted in triplicate and three independent assays were performed. Standard error of the mean is shown.

Several investigators have identified Rta responsive elements in viral promoters for both KSHV and MHV68 [40]–[44]. To date, the known Rta responsive genes are either regulated through: (i) direct interaction of Rta and DNA through a core Rta binding sequence; or (ii) Rta DNA binding is facilitated through protein-protein interactions – in the case of KSHV Rta, through interaction with the cellular transcription factor RBPJκ (reviewed in [45]). In MHV68 gene 57 promoter, it appears that both types of Rta response elements may be utilized – although a role for RBPJκ in MHV68 Rta activation has not been formally demonstrated [40], [41]. Interestingly, neither of the binding sites identified in the MHV68 gene 57 promoter are present in the M1 promoter, suggesting a novel Rta interaction motif. To identify the Rta response element(s) in the M1 promoter, a series of promoter truncations were generated and tested in the P3X63Ag8 plasmacytoma cell line. A candidate Rta response element was identified by evaluating promoter constructs which lost the ability to be transactivated by Rta. Using this approach we identified a putative Rta response element between −82 and −72 bp in the M1 promoter (Figure 9A). This 12 bp sequence (5′-GGTCAGAAGGCT-3′) failed to show homology to any known Rta response element identified in the gammaherpesvirus family. However, a screen of the MHV68 genome identified a number of candidate sites upstream of other MHV68 replication-associated genes which share significant homology with the core 5′-TCAGAAG-3′ sequence in the putative M1 promoter Rta response element (Figure 9B). Mutations of the three most central residues of the predicted Rta response element (see M1pRREm in Figure 9B) resulted in an ca. 10-fold reduction in transactivation in the plasma cell line (Figure 9C), as well as an ca. 6-fold reduction in Rta and IRF4 synergistic transactivation of the M1 promoter in 293T cells (Figure 9D).

### The novel Rta response element in the M1 promoter is conserved in other viral promoters

**Figure 10:**
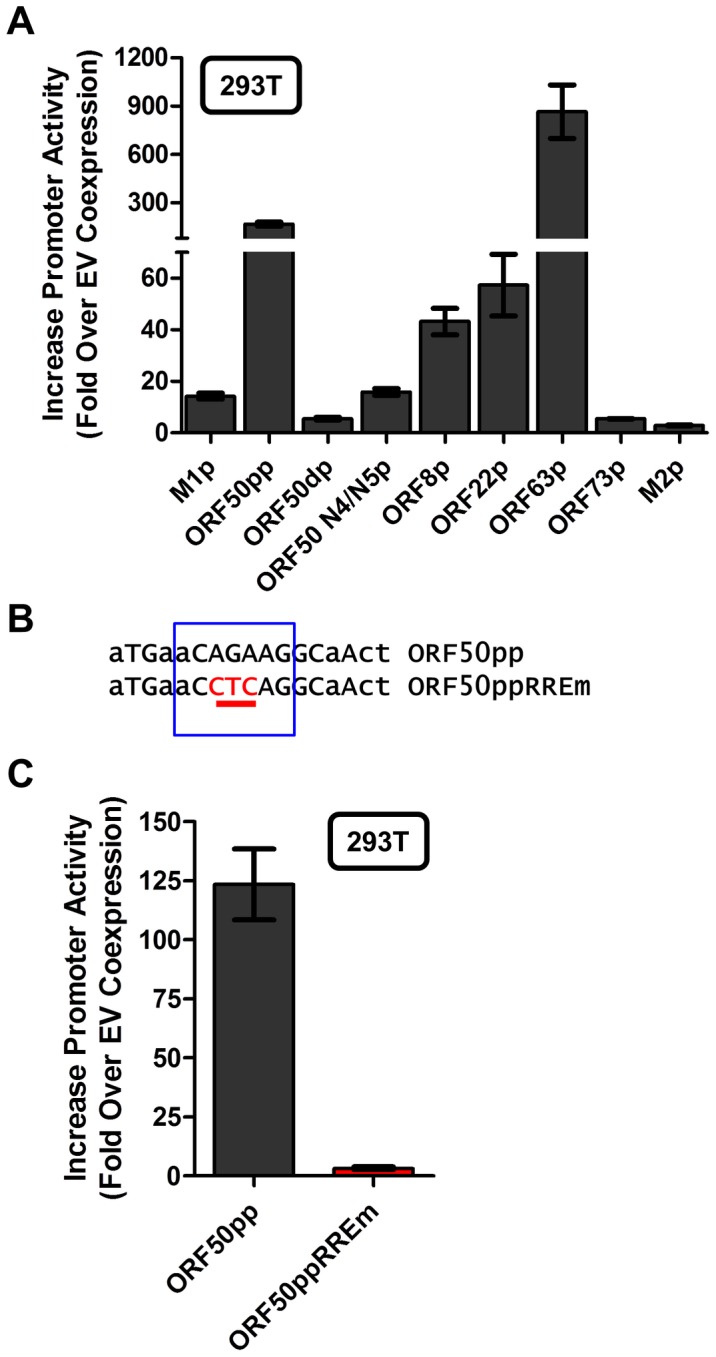
Novel RRE involved in Rta activation of the gene 50 proximal promoter. (A) Analysis of Rta transactivation of MHV68 promoters that contain the core sequence present in the novel RRE identified in the M1 promoter. The indicated promoter-driven luciferase reporter constructs were transfected into 293T cells in the presence and absence of an Rta expression plasmid. (B) Mutation introduced into the putative RRE present in the gene 50 proximal promoter (ORF50pp described in [46]). (C) Analysis of Rta transactivation of the wt and RRE mutant gene 50 proximal promoter transfected into 293T cells.

With the identification of a novel Rta response element, we next wanted to evaluate whether this element was functional in other viral promoters that appear to contain this RRE (see Fig. 9C). Reporter constructs for the putative promoter regions of the M2 gene (encoding an adaptor protein involved in B cell signaling), ORF8 (encoding glycoprotein B), ORF22 (encoding glycoprotein H), ORF63 (encoding a tegument protein), and ORF73 (encoding the MHV68 Latency Associated Nuclear Antigen (LANA) homolog) were generated. In addition, the gene 50 proximal, distal, and N4/N5 promoter constructs previously described in Wakeman *et al.* [46] were evaluated for response to Rta expression. We observed varying levels of promoter response, with the strongest responses from ORF50pp, ORF8p, ORF22p, ORF63p, intermediate responses from the M1p, ORF50dp and ORF50 N4/N5p, and weak responses from M2p and ORF73p (Figure 10A). To further investigate the role of the Rta response element in the observed transactivation, we engineered the same three nucleotide mutation used in the M1p (Figure 9B) into the proximal ORF50 promoter (Figure 10B). Notably, mutation of this sequence resulted in a 38-fold reduction in Rta transactivation (Figure 10C). Notably, with the exception of the M1 promoter, for all the other reporter construct we failed to observe any synergistic activation by the co-expression of Rta and IRF4 (data not shown).

## Discussion

Here we described the characterization of a recombinant MHV68 in which a gene encoding a fluorescent protein (YFP) has been introduced into the viral genome in place of a non-essential viral gene. This approach allows identification of the site and timing of viral gene expression *in vivo* for viral genes that are dispensable for replication and/or dissemination of virus. For viral genes that play an important role in either replication or dissemination, other approaches - such as the generation of fusion gene products - may be required. Information obtained from such studies can provide significant insights into viral gene function and their mode of action. In the case of M1, these analyses led to identification of the predominant cellular reservoir in which M1 is expressed, and subsequent identification of transcription factors involved in regulating M1 gene transcription.

Coppola *et al.* have previously demonstrated the ability of either B220+ cells, or T cell depleted splenocytes, isolated from MHV68 infected mouse spleen to stimulate Vβ4^+^ CD8^+^ T cell hybridomas [11]. However, they also found that B cell depleted splenocytes from MHV68 infected mice retained Vβ4^+^ CD8^+^ T cell stimulatory activity – which they interpreted as the presence of other non-B cells populations in the spleen that are infected by MHV68. However, based on our findings that M1 expression is largely restricted to plasma cells, it seems unlikely that either the isolation or depletion of B220+ cells would efficiently capture or eliminate, respectively, all MHV68 infected plasma cells. As such, one would anticipate Vβ4^+^ CD8^+^ T cell stimulatory activity in both the enriched and depleted fractions. This interpretation is consistent with the complete failure to observe any expansion of Vβ4^+^ CD8^+^ T cells in MHV68 infected B cell-deficient mice [9], [10] – even though we have previously shown robust MHV68 infection in the spleens of B cell-deficient mice under some experimental conditions (intraperitoneal inoculation of virus) in the absence of any detectable Vβ4^+^ CD8^+^ T cell expansion [10], [47].

As we have previously shown, MHV68 reactivation in the spleen is tightly linked to plasma cell differentiation [27]. The observation that M1 is predominantly expressed in plasma cells thus suggested that M1 expression is linked to virus reactivation/replication. This was substantiated by demonstration that Rta can strongly transactivate the M1 promoter in a plasma cell line (see Figure 8A). We propose that during infection, in response to viral reactivation and the transition from germinal center or memory B cell to plasma cell, M1 expression is activated by the synergistic effects of viral Rta and cellular IRF4. M1 protein is secreted from infected plasma cells and, by an undefined mechanism, stimulates Vβ4^+^ CD8^+^ T cell activation and expansion. It is likely that M1 activates Vβ4^+^ CD8^+^ T cells via a mechanism similar to classic viral super-antigens [2]. Activation does not require classical MHC class I molecules [11], [48], but does require an intact M1 protein - we have previously shown that proteolytic digestion, or denaturation of recombinant M1 renders it unable to activate Vβ4^+^ CD8^+^ T cell hybridomas [2].

Vβ4^+^ CD8^+^ T cells have been shown to traffic throughout the body, and can be detected in the blood, spleen, liver, lung, and peritoneal cavity [2], [3], [8]. These cells show cytolytic activity [8] and adopt an effector memory phenotype where upon re-stimulation with recombinant M1 protein *ex vivo* they degranulate and produce INFγ and TNF α ([2], unpublished observations). As IFNγ has been shown to regulate MHV68 reactivation from macrophages in the peritoneum, but not reactivation from splenic B cells [6], we would predict that the Vβ4^+^ CD8^+^ T cells traffic to sites in which infection is less tightly controlled, to suppress MHV68 reactivation through the secretion of IFNγ in a paracrine fashion. We find it noteworthy that MHV68 M1-null infected mice exhibit hyper-reactivation in the peritoneal cavity and persistent viral replication in the lung [1], [2], [49], further underscoring the importance of M1 expression in controlling viral infection.

**Figure 11:**
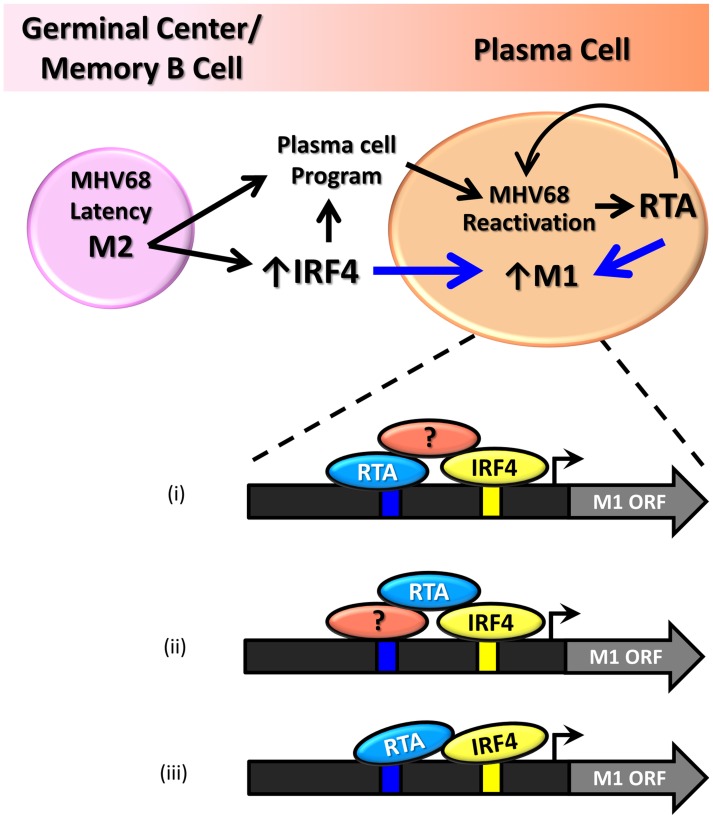
Model of M1 gene regulation upon differentiation of latently infected B cells to plasma cells and ensuing virus reactivation. During infection MHV68 M2 regulates the expression of cellular IRF4, a critical transcription factor in plasma cell differentiation. M2 has also been shown to be critical for viral reactivation in the plasma cell reservoir. Increased levels of Rta and IR4 during viral reactivation in infected plasma cells lead to increased expression of M1, which is mediated through protein:protein interactions between Rta and IRF4, as well as binding through cis-elements within the M1 promoter. Shown are 3 possible mechanism for how Rta synergizes with IRF4 to activate transcription of the M1 gene: (i) Rta binds to the novel RRE and interaction with IRF4 is mediated by a cellular and/or viral factor that binds both Rta and IRF4; (ii) Rta interacts with a cellular and/or viral factor bound to the RRE and also directly binds to IRF4; and (iii) Rta binds to the RRE and interacts directly with IRF4.

Our findings demonstrate that the M1 promoter is regulated by MHV68 Rta, a viral transcription factor that is essential for induction of viral reactivation. Rta activation of the M1 promoter synergizes with IRF4, a transcription factor that plays a critical role in both plasma cell differentiation and immunoglobulin class switch recombination. Furthermore, we show that this interaction is likely mediated through both DNA-protein interactions with the M1 promoter sequence, as well as protein-protein interactions between Rta and IRF4. We propose that during MHV68 latency, the viral latency-associated gene product M2 is expressed in a sub-population of latently infected germinal center and memory B cells [21] leading to expression of high levels of IRF4 [50]. M2 appears to play an important role in virus reactivation from latency: (i) MHV68 M2 null mutants exhibit a profound reactivation defect from B cells, but not latently infected macrophages [51], [52]; (ii) exogenous expression of M2 in primary B cells results in acquisition of a pre-plasma memory phenotype [53]; (iii) M2 can drive B cell differentiation of a B lymphoma cell line *in vitro* [27]; (iv) M2 is required for efficient immunoglobulin class switch in infected B cells *in vivo* [27]; and (v) plasma cells are the primary source of viral reactivation from the spleen [27]. Taken together these data suggest that MHV68 is capable of driving plasma cell differentiation, and concurrent with this differentiation, viral reactivation. As a result of this transition, the increased expression of the transcription factors Rta and IRF4 lead to induction of M1 expression in plasma cells (Figure 11).

That M1 is responsive to viral Rta and cellular IRF4 highlights the importance of tightly regulated gene expression in response to host and viral cues. This promotes cell type specific expression coordinated with viral reactivation. Furthermore, the interaction with Rta and IRF4 suggests a conserved strategy for gene regulation in MHV68, allowing for better control of Rta responsive gene expression. In fact numerous lytic genes in MHV68 appear to share the Rta response element identified in the M1 promoter (Figure 9B).

Though our efforts to find other viral genes that are similarly responsive to the concerted effects of Rta and IRF4 were unsuccessful, we find it attractive to speculate that the partnership of Rta and IRF4 or other cellular transcription factors may mediate their gene expression in a cell type specific manner. However, many of the genes we evaluated showed response to the novel Rta response element. Our analysis was limited to the putative promoter regions of ORF50, ORF8, ORF22, ORF63, ORF73, and M2. However, many of these genes play critical role in the biology of the virus, either as structural genes- ORF8 and ORF 22 are both surface glycoproteins, or as genes involved initiating infection- ORF63 is a tegument protein; so it is not surprising to find a significant response to Rta but lack of synergy with IRF4. Furthermore, some of the candidate genes are known to be involved in viral latency, ORF73- or murine latency associated nuclear antigen (mLANA) a homolog of EBV and KSHV LANA, has many functions including: viral replication, episomal maintenance, transcriptional regulation, and dysregulation of cell cycle and cell division (reviewed in [54]). M2, a latency associate protein appears to play roles in both maintenance and establishment of latency, as well as in viral reactivation [50], [53], [55]. We therefore find it plausible that these genes would have less stringent requirements for cell specific expression, and that other unidentified genes, might be regulated by Rta and IRF4. Additionally, due to the differing functions of these genes in MHV68 biology, we were not surprised that in our studies we found differing levels of Rta responsiveness. Future studies using genome wide analyses will be necessary to identify genes which are temporally regulated by viral and host factors including Rta and IRF4.

Our identification of a partnered interaction between Rta and IRF4 suggests a conserved method for regulating MHV68 viral gene expression. Moreover, this mechanism appears throughout the gammaherpesviruses family as several studies have shown that Rta is capable of binding DNA through orchestration of complex protein-protein interactions. In KSHV, kRta has been shown to directly interact with cellular Oct1 and RBPJκ to regulate the KSHV bZip promoter [43]. This interaction with RBPJκ is maintained through a tetrameric protein complex of kRta flanking RBPJκ, and is mediated through a core “CANT” DNA repeat element found in the Mta promoter sequence [42]. Notably, kRTA has also been found to interact with viral IRF4 (vIRF4), one of several viral IRF homologs encoded by KSHV which in the case of vIRF4 is involved in counteracting innate antiviral defenses mediated by interferons to regulate vIRF1, vIRF4, PAN, and ORF57 gene expression [44].

In summary, the data reported here defines the timing and location of M1 expression during *in vivo* infection using a recombinant reporter virus – demonstrating that M1 is predominantly expressed from plasma cells. Furthermore, M1 gene transcription in plasma cells is driven by the viral immediate-early Rta in conjunction with cellular IRF4 – which potently synergize with each other to activate M1 gene transcription. Whether other viral (and perhaps cellular genes) are co-regulated by Rta and IRF4 remains to be determined, and will be the topic of future work. However, we find it interesting to speculate that this might be an effective strategy to target viral replication-associated gene expression in plasma cells.

## Methods

### Ethics statement

This study was carried out in strict accordance with the recommendations in the Guide for the Care and Use of Laboratory Animals of the National Institutes of Health. The protocol was approved by the Emory University Institutional Animal Care and Use Committee and in accordance with established guidelines and policies at Emory University School of Medicine (Protocol Number: YER-2002245-031416GN).

### Mice and virus infections

Six to eight week old female C57Bl/6 mice were obtained through Jackson Laboratory (Bar Harbor, ME) and housed at Emory University in accordance with university guidelines. Prior to infection mice were sedated with isofluorane and intranasally infected with 5×10^5^ pfu in 20 ul of DMEM.

### Cell lines

Cells were grown under normal conditions at 37°C with 5% CO2. A20-HE2 cell were grown in complete RPMI-1640 (supplemented with 10% FCS, 100 U/mL penicillin, 100 mg/mL streptomycin, 2 mM L-glutamine, and 50 mM β-mercaptoethanol); P3X63Ag8 (ATCC TIB-9) were grown in compete RPMI-1640 with the addition of 10 mM non-essential amino acids, 1 mM sodium pyruvate, and 10 mM HEPES; and 293T cells (a generous gift from Dr. Edward Mocarski) were grown in complete DMEM (supplemented with 10% FCS, 100 U/mL penicillin, 100 mg/mL streptomycin, and 2 mM L-glutamine).

### Generation of recombinant viruses

To generate the M1 promoter driven YFP virus a 500 bp homology arm immediately upstream of M1 ORF was amplified with LFA_MluI_1521-1573 (5′-TCCCCAATGACGCCAAAGTCTAAGTCCCTGTACAGGCTTAACTTTTTTAGAAT-3′) and LFA_SpeI_2005-2022 (5′-GGTCGCCGCTGCTCAATG-3′) and cloned into pCR Blunt-eYFP vector (a kind gift from Dr. Chris Collins) using MluI and SpeI to generate pCR Blunt-eYFP M1 LFA Flank. Next a 495 bp homology arm immediately downstream of the M1 ORF was PCR amplified using RFA_NotI_3286-3307 (5′- GCCTGAATACATGTTTACTGGG-3′) and RFA_NsiI_3758-3780 (5′- AACCTACGCGGCCACTCAACAGA -3′) was cloned into pCR Blunt-eYFP using NotI and NsiI to create pCR Blunt-eYFP M1 LFA RFA flank. The eYFP flanked by left and right homology arms for the M1 locus was then PCR amplified to include BglII and NsiI restriction sites and was cloned into pGS284. The resulting plasmid pGS284-eYFP M1 LFA RFA flank was then electroporated into λPir electro-competent bacteria for allelic exchange with WT MHV68 BAC in GS500 RecA+ *Escherichia coli*. To generate the M1st-eYFP virus, GS500 containing M1st. BAC [2] were crossed with λPir containing pGS284-XL9CD-CMV-YFP-F for allelic exchange. Following allelic exchange virus preparation was performed as previously described [23].

### Flow cytometry

Single cell suspensions of splenocytes were prepared and resuspended in PBS supplemented with 2% fetal bovine serum. Samples were stained using standard procedures. Following initial FC receptor block (CD16/32), samples were stained with a master mix containing: CD138-PE, CD3e-PerCP, CD95-PE, GL7-APC, B220-APC-Cy7, CD19-Pacific Blue. 1–2×10^6^ events were recorded on BD LSRII flow cytometer and results were analyzed using FloJo software (Tree Star Inc).

### Rapid amplification of cDNA ends (RACE) analysis

A20-HE2 cells were stimulated with 20 ng/mL tetradecanoylphorbol acetate (TPA) for 48 hours prior to RNA isolation. NIH3T12 were infected with an MOI of 1.5 for 18 hours prior to RNA isolation. RNA was extracted using Trizol Reagent (Invitrogen Life Technologies) according to manufacturer's instructions and RNA concentration was determined. Prior to RACE analysis RT-PCR was performed to detect M1 and pol transcripts using primers described previously [56]. 5′ and 3′ RACE analysis was performed using GeneRacer Kit L1502-02 (Invitrogen Life Technologies) according to manufacturer's specifications. Gene specific primers were generated for detection of M1 transcript. For the first round of PCR M1ORF_Rd1_Fwd (5′-GGCCATTATGTGGACGTGAAGAGAATTGTAGGTAT-3′) was used to amplify the 3′ region and M1ORF_Rd1_Rvs (5′-CCTTGGTATCATCCTCAGGAAATGGGTAGGTTTCA-3′) was used to amplify the 5′ region. For the second round of PCR M1ORF_Rd2_Fwd (5′-GGAAAACTCTCCAGAGCTGCTGTCGTG GGGGATGAT-3′) was used to amplify the 3′ region and M1ORF_Rd2_Rvs (5′-GCCAGTGAGCTATGCTTTGGCCCAGTATGCAGGAA-3′) was used to amplify the 5′ region.

### Generation of plasmids

M1 promoter luciferase constructs were cloned into pGL4.10 (Promega) using BglII and KpnI restriction sites. With the exception of the M1pIRF4mut1, M1pIRF4mut2, and G50ppRREm binding site mutants, inserts were generated by PCR amplification of regions upstream of the M1 ORF, using WT BAC DNA as template, with primers listed in table 1. The M2 promoter construct was the generous gift from Shariya Terrell.

**Table 1:** Oligonucleotide primer sequences.

**M1p 1025 bp–forward**	gatcggtacccttccagctaagatagcatgtgccg
**M1p 1025 bp–reverse**	ctagagatctggtcgccgctgctcaatgatg
**M1p 525 bp–forward**	gctcggtacccccagaagaccatgtctgggaat
**M1p 525 bp–reverse**	gctagatctggtcgccgctgctcaatg
**M1p 245 bp–forward**	gcgggtacccctaccccttttgctccac
**M1p 245 bp–reverse**	gctagatctggtcgccgctgctcaatg
**M1p 197 bp–forward**	gcgggtacccaaaacaagaacagttgc
**M1p 197 bp–reverse**	gctagatctggtcgccgctgctcaatg
**M1p 100 bp–forward**	gcgggtaccgtttcaaacactggtcag
**M1p 100 bp–reverse**	gctagatctggtcgccgctgctcaatg
**M1p 100 bp RREm–forward**	gcgggtaccgtttcaaacactggtcctcaggctatc
**M1p 100 bp RREm–reverse**	gctagatctggtcgccgctgctcaatg
**M1p 88 bp–forward**	gcgggtaacggtcataaggctatc
**M1p 88 bp–reverse**	gctagatctggtcgccgctgctcaatg
**M1p 82 bp–forward**	gccggtaccaaggctatctttctt
**M1p 82 bp–reverse**	gctagatctggtcgccgctgctcaatg
**M1p 76 bp–forward**	gcgggtaccatctttcttggtggt
**M1p 76 bp–reverse**	gctagatctggtcgccgctgctcaatg
**M1p 64 bp–forward**	gcgggtaccggtttcacttctaaa
**M1p 64 bp–reverse**	gctagatctggtcgccgctgctcaatg
**M1p unspliced Rta–forward**	ggcgcggccgcatggcctctgactcggattccc
**M1p unspliced Rta –reverse**	ggcctcg ggcctcgagttatgactccaggctgtttggg
**M1p unsplice C-flag RTA–forward**	ggcgcggccgcatggcctctgactcggattccc
**M1p unspliced C-flag RTa–reverse**	cggctcgagctacttatcgtcgtcatccttgtaatctgactccaggctgtttg
**M1p mIRF4–forward**	ggcgcggccgcatgaccttggagacgggcagc
**M1p mIRF4–reverse**	ggcctcgagtcactcttggatggaagaatgac
**M1p ORF8p–forward**	ccgggtaccccaaaattgttgcggccctaag
**M1p ORF8p–reverse**	gccagatctcaacactggctgaatcggttc
**M1p ORF22p–forward**	ccgggtaacctgcgacccgagtcaaggagag
**M1p ORF22p–reverse**	gccagatctttgtgcttggctatttattactac
**M1p ORF63p–forward**	ccgctcgagcagccagcacattagcttcatgg
**M1p ORF63p–reverse**	gccagatcttatgtcagaagttggtaaatat
**M1p ORF73p–forward**	ccgggtaccataggacaatgatgagttttc
**M1p ORF73p–reverse**	gccctcg gccctcgagtatctgaaagagataaagtacac
**M1p 50 sense**	gctgggtaccacatgggccattaaaagggagggaattggcatcattgagcagcggcgaccagatctgtcg
**M1p 50 antisense**	cgacagatctggtcgccgctgctcaatgatgccaattccctcccttttaatggcccatgtggtacccagc
**M1p 25 sense**	gctgggtaccttggcatcattgagcagcggcgaccagatctgtcg
**M1p 25 antisense**	cgacagatctggtcgccgctgctcaatgatgccaaggtacccagc

To generate M1pIRF4mut1 and 2 Overlapping PCR was used to introduce IRF4/IRF8 binding site mutations into the 197 bp M1 promoter region corresponding to nt. 1960–1961 and nt. 1961–1963 in the viral for mutants 1 and 2 respectively. Amplification of a 118 bp left flaking arm was done using 197 bp forward primer (table 1) and reverse primers: (5′-TCTTTCTTGGTGTGTTCACTTCTAAACATG-3′) and (5′-TCTTTCTTGGTGGGACCACTTCTAAACATG -3′) for mutants 1 and 2 respectively. Amplification of a 70 bp right flanking arm was done using 197 bp reverse primer (table 1) and forward primers: (5′-CATGTTTAGAAGTGAACACACCAAGAAAGA-3′) and (5′-CATGTTTAGAAGTGGTCCCACCAAGAAAGA-3′) for mutants 1 and 2 respectively. The left and right flanking arms were used as template and allowed to anneal for 6 rounds of the PCR cycle prior to the addition of the 197 bp forward and reverse primers. The resulting amplicon was then cloned into pGL4.10.

To generate the ORF50ppRREm MHV68 WT BAC DNA was used as a template for overlapping PCR. In the first PCR round left and right flanking arms were generated using ProxPromF (5′-GATCGCTAGCTCTTTATAGGTACCAGGGAA-3′) with ProxRREmR (5′-tcactctgttcaagaagttgcctgaggttcataaa-3′), and ProxPromR (5′-TAGCAGATCTGGTCACATCTGACAGAGAAA-3′) with ProxRREmF (5′-ttcattttcaggccatttatgaacctcaggcaact-3′) respectively. These products were used as a template for a second round PCR amplification with primers ProxPromF and ProxPromR, and amplicons were cloned into pGL4.10.

Expression constructs were cloned into pCDNA 3.1 (+) (Invitrogen) using NotI and XhoI restriction sites using primers listed in table 1. Both flag-tagged and non-tagged unspliced Rta were amplified from WT BAC DNA corresponding to viral genomic coordinates (66,761–69,374). Murine IRF4 was amplified from pMSCV-IRF4-IRES-GFP (a kind gift from Dr. Xiaozhen Liang).

All PCR amplification was carried out using high fidelity Phusion DNA polymerase (New England Biolabs), and sequence analysis confirmed completed plasmid constructs (Macrogen).

### *In vitro* promoter assays

5×10^5^ 293T cells were seeded into 6 well plates, the following day cells were transfected with 2.5 ug firefly luciferase and protein expression plasmids and 10 ng of pRL-TK (Promega) using TransIT 293T (Mirus). 1×10^6^ P3X63Ag8 cells were nucleofected with 5 ug firefly luciferase plasmids using Ingenio Electroporation Solution (Mirus) using setting X-01 on the Amaxa nucleofector. Reactions were done in triplicate for each condition, and 2–4 independent experiments were conducted. 48 hours later cells were lysed using passive lysis buffer (25 mM Tris-phosphate pH 7.8, 2 mM DTT, 2 mM DCTA, 10% glycerol, 1% Triton X-100). P3X63Ag8 cells were assessed for firefly luciferase activity using 10 µl lysate and 50 µl luciferase assay reagent (LAR) (75 mM HEPES pH 8, 4 mM MgSO4, 20 mM DTT, 100 µM EDTA, 53.0 µM ATP, 270 µM Coenzyme A, and 470 µM beetle Luciferin). A dual luciferase assay for firefly and renilla luciferase activity was performed on 293T cells using 10 µl cell lysate and 50 µl LAR, followed by the addition of 50 µl Stop & Glo reagent (Promega). Light units were read on a TD-20/20 luminometer.

### Nuclear extract and electrophoretic mobility shift assay

Nuclear extracts of P3X63Ag8 cells grown under normal conditions were made as previously described [57]. Briefly, cells were washed with PBS, pelleted cells, resuspended in ice cold hypotonic lysis buffer and incubated on ice for 15 minutes. 10% Nonidet P-40 was added at 1/20 final volume and nuclei were spun down. Nuclei were then washed in hypotonic lysis buffer, resuspended in high salt buffer, and incubated with vigorous shaking for 2–3 hours at 4°C. Supernatants were collected following centrifugation and aliquoted on dry ice and stored at −80°C. Following isolation protein content in nuclear extract was quantified using DC Protein Assay (BioRad), and western blot was performed to confirm presence of IRF4.

Electrophoretic mobility shift assay was performed using nuclear extracts as previously described [58]. Briefly, a binding reaction containing 10 µg of nuclear extract, 0.2 ng ^32^P-labeled double stranded oligonucleotide probe containing IRF4 consensus binding sequence (underlined) (sense-5′-TTGGTGGTTTCACTTCTAAACA-3′), and 2 ug poly (di-dC) was made up in binding buffer (10 mM Tris-HCl (pH 7.5), 10 mM HEPES, 50 mM KCl, 1.1 mM EDTA, and 15% glycerol, with 1.25 mM DTT) and incubated on ice for 30 minutes. Supershift assays included 1 ug of IRF4 antibody (clone M17, Santa Cruz Biotech.) or isotype control pSTAT1 antibody (clone Tyr 701, Santa Cruz Biotech.) incubated with nuclear extracts slow shaking at 4°C for 1 hour. Competition experiments were performed with 2X and 20X unlabeled oligonucleotides containing WT or mutated (underlined) IRF4 consensus binding sequence (sense 5′-TTGGTGGGACCACTTCTAAACA-3′). Nucleoprotein complexes were run on 5% native polyacrylamide gel in 0.5X Tris Buffered EDTA at 180 V for 1 hour. Gel was dried under vacuum and analyzed with PhosphorImager analysis (Typhoon 9410; Amerisham Bioscience).

### Co-immunoprecipitation

10 cm dishes were seeded with 4×10^6^ 293T and were transfected the next day using TransIT-293T (Mirus). 48 hours later cells were washed 2 times with ice cold PBS, and lysed while rocking at 4°C, in 1 mL Triton X Lysis Buffer (50 mM Tris HCl pH 7.4–7.5, 150 mM NaCl, 1 mM EDTA, 0.1% Triton; supplemented with 1 mM NaF, 1 mM activated Na3V04, and Roche EDTA free protease inhibitor cocktail tablet for 50 mL volume). Following lysis, membranes were pelleted and lysates were transferred to pre-chilled tubes. Protein concentration was determined using DC Protein Assay (BioRad). 1 mg of cell lysate was precleared with prepared protein G beads (Pierce), then 8 ug of IRF4 antibody (clone M17, Santa Cruz Biotech) or flag antibody (clone M2, Sigma) was added and lysates were incubated overnight at 4°C. Lysates were transferred to freshly prepared protein G beads for binding and were incubated at 4°C for 2 hours. Following wash, protein was eluted and samples were electrophoresed on 10% polyacrylamide gels, and transferred onto nitrocellulose membranes for western blot. The following detection antibodies were used: IRF4 (clone H140, Santa Cruz Biotech.) and Flag (clone M2, Sigma).
